# Insight into the Adsorption Behaviors of Antimony onto Soils Using Multidisciplinary Characterization

**DOI:** 10.3390/ijerph19074254

**Published:** 2022-04-02

**Authors:** Zi-Qi Mu, Da-Mao Xu, Rong-Bing Fu

**Affiliations:** 1 State Key Laboratory of Pollution Control and Resource Reuse, College of Environmental Science and Engineering, Tongji University, Shanghai 200092, China; 1930521@tongji.edu.cn; 2 Centre for Environmental Risk Management and Remediation of Soil and Groundwater, Tongji University, Shanghai 200092, China; 3 Shanghai Institute of Pollution Control and Ecological Security, Shanghai 200092, China

**Keywords:** antimony pollution, soil, adsorption

## Abstract

Antimony (Sb) pollution in soils is an important environmental problem, and it is imperative to investigate the migration and transformation behavior of Sb in soils. The adsorption behaviors and interaction mechanisms of Sb in soils were studied using integrated characterization techniques and the batch equilibrium method. The results indicated that the adsorption kinetics and isotherms of Sb onto soils were well fitted by the first-order kinetic, Langmuir, and Freundlich models, respectively, while the maximum adsorbed amounts of Sb (III) in soil 1 and soil 2 were 1314.46 mg/kg and 1359.25 mg/kg, respectively, and those of Sb (V) in soil 1 and soil 2 were 415.65 mg/kg and 535.97 mg/kg, respectively. In addition, pH ranging from 4 to 10 had little effect on the adsorption behavior of Sb. Moreover, it was found that Sb was mainly present in the residue fractions, indicating that Sb had high geochemical stability in soils. SEM analysis indicated that the distribution positions of Sb were highly coincident with Ca, which was mainly due to the existence of calcium oxides, such as calcium carbonate and calcium hydroxide, that affected Sb adsorption, and further resulted in Sb and Ca bearing co-precipitation. XPS analysis revealed the valence state transformation of Sb (III) and Sb (V), suggesting that Fe/Mn oxides and reactive oxygen species (ROS) served as oxidant or reductant to promote the occurrence of the Sb redox reaction. Sb was mobile and leachable in soils and posed a significant threat to surface soils, organisms, and groundwater. This work provides a fundamental understanding of Sb adsorption onto soils, as well as a theoretical guide for studies on the adsorption and migration behavior of Sb in soils.

## 1. Introduction

Antimony (Sb) pollution is becoming a global environmental issue because of its significant environmental hazards to animals, plants, microorganisms, and humans [1,2,3]. It is shown that Sb can inhibit the growth of microorganisms and enzyme activities in soils, while it has negative impacts on the growth and proliferation of bacteria, fungi, actinomycetes, and some sensitive enzymes in soils [4,5,6]. Sb is easily absorbed by human spleen and blood cells before being accumulated in vascularized organs and tissues, which results in serious health risks to humans, such as respiratory diseases, cardiovascular diseases, and liver diseases [7,8]. As a carcinogenic element, Sb is now classified as an emerging pollutant by the United States Environment Protection Agency (US EPA) and European Union (EU), as a result of various anthropogenic activities, including mining, smelting, coal combustion, and other industrial activities [9,10,11]. In addition, Sb pollution is of particular environmental concern in many countries, including the USA, Australia, China, Japan, Mexico, New Zealand, Spain, and Slovakia [12,13,14,15,16]. To date, China is the largest emitter and producer of Sb globally. It is estimated that in 2010 alone, China discharged around 649 t of Sb, subsequently contributing to more than 60% of global Sb production in 2019 [17]. In nature, Sb concentrations in soils are generally between <1 and 4 mg/kg [18]. In some cases, elevated Sb concentrations in soils are over 1500 mg/kg [19].

Sb is primarily presented as Sb (OH)_3_ and Sb (OH)_6_ in soils, respectively [20,21]. It is widely reported that Sb mainly exists in two inorganic oxidation states of Sb (III) and Sb (V) [22,23]. It is also shown that Sb (V) dominates in oxidizing conditions, mostly in the soils with the shallow layer, whereas Sb (III) is dominant in anoxic conditions, especially in the deeper saturated zones [23]. In soils and groundwater, Sb appears to be the predominant form of Sb (V) [24,25]. In contrast, Sb (III) is the most toxic form, which can be quickly and non-reversibly absorbed onto soil minerals, such as organic matter and carbonates in alkaline soils [26,27], and it can also be oxidized to Sb (V) at more negative Eh values [28].

Furthermore, many studies have showed that the speciation, mobility and availability of Sb in soils are strongly affected by soil physicochemical properties, such as pH, cation-exchange capacity (CEC), soil texture, inorganic and organic ligands [29]. Among these properties, pH can affect the chemical speciation and ionization degree of trace metals [30,31]. In addition, the migration, transformation, and bioavailability of Sb in soils are related to its forms, adsorption state, and soil properties [19]. Sb mainly exists in soils in the form of low-soluble sulfides. Meanwhile, it is also easily associated with Fe and Al oxides or organic matter in soils, which decreases its migration ability [32,33,34].

Most studies have focused on the adsorption of Sb on various geotechnical minerals and adsorbents [34,35,36], while the migration and transformation of Sb in soil and groundwater systems have rarely been studied. In addition, the migration and transformation mechanisms of Sb in the soil and groundwater system remain unknown. In this study, Sb (III) and Sb (V) solution are considered as the polluted groundwater, and soils collected from a Shanghai aquifer are used as adsorbents. A comparison of the adsorption behaviors of Sb (III) and Sb (V) onto soils was conducted to explore the migration and transformation of Sb in the soil and groundwater system. This work might be helpful for providing a novel insight into the geochemical behaviors of Sb in the system and its risk management.

## 2. Materials and Methods

### 2.1. Experimental Materials

#### 2.1.1. Chemical Reagents

Potassium pyroantimonate (K_2_H_2_Sb_2_O_7_), potassium antimony tartrate (K(SbO)C_4_H_4_O_6_·1/2H_2_O), sodium hydroxide (NaOH), hydrochloric acid (HCl), and potassium chloride (KCl) were provided by Sinopharm Chemical Reagent Company (Shanghai, China). All solutions were prepared with ultrapure water (Shanghai, China).

#### 2.1.2. Soil Samples

Two shallow soil samples were collected from one site in Shanghai, China (31°10′46″ N, 121°26′26.01″ E). In the soil layer structures of the site, two soil samples were adjacent to each other, i.e., soil 1 was located on soil 2. Studies on the adsorption of two soils can help to clarify the migration of Sb between different soil textures. The soil samples were naturally dried at room temperature, homogenized, ground manually, and passed through a 0.2-mm mesh sieve. The physical and chemical properties of soils are shown in Table 1.

### 2.2. Adsorption Experiments

#### 2.2.1. Kinetic Experiment

Briefly, 200 mg/L Sb (III) and Sb (V) solutions were prepared at room temperature (25 °C). The ionic strength was adjusted to 0.01 mol/L with KCl solution, because KCl did not react with Sb solutions and existed in the natural groundwater. Then, the pH of the solution was adjusted to 4 with NaOH and HCl to simulate groundwater conditions at contaminated sites. Hence, 2.0000 g of soils were added to a 50 mL plastic centrifuge tube. To control soil/water ratio at 1:10, 20 mL of the solution was added to the centrifuge tube, and the samples were shaken at a constant temperature, respectively. After 0.17, 0.33, 0.5, 1, 3, 5, 8, 24, 36, 48, 72, and 120 h shaking, the centrifuge tubes were taken out and centrifuged at 4000 r/min for 20 min. After filtration, Sb concentrations in the filtrations were determined using ICP-OES (Agilent, Palo Alto, CA, USA).

The experimental results were fitted using the first-order kinetic model and the second-order kinetic model to study the adsorption mechanism of Sb (III) and Sb (V) on soils in Equations (1) and (2), which are given below [37,38]:

(1) $$Q_{t}=Q_{e}\left(1-e^{-k_{1}x}\right)$$

(2) $$Q_{t}=\frac{Q_{e}{}^{2}k_{2}x}{1+Q_{e}k_{2}x}$$

where Q_t_ (mg/kg) and Q_e_ (mg/kg) are the adsorption capacity at time t and equilibrium, respectively; k_1_ (h^−1^), k_2_ (kg/(mg·h)) are the rates of the first-order kinetics and the second-order kinetics, respectively; t (h) is the adsorption time.

#### 2.2.2. Isothermal Experiment

Briefly, 1, 5, 10, 30, 50, and 100 mg/L Sb (III) and Sb (V) solutions were prepared at room temperature (25 °C). Then, 2.0000 g of soils were added to a 50 mL plastic centrifuge tube. The pH adjustment, KCl concentration, soil/water ratio, and shaking time were the same as in Section 2.2.1.

Langmuir and Freundlich models were used to fit the isotherm adsorption experimental results.

The Langmuir isotherm adsorption model is:

(3) $$Q_{e}=\frac{Q_{m}K_{L}C_{e}}{1+K_{L}C_{e}}$$

where Q_m_ (mg/kg) is the maximum adsorption capacity; K_L_ (L/kg) is the adsorption equilibrium constant; Q_e_ (mg/kg) and C_e_ (mg/L) are the adsorption capacity on the solid phase and the equilibrium concentration in the suspension.

The Freundlich isotherm adsorption model is:

(4) $$Q_{e}=K_{F}C_{e}^{1/n_{F}}$$

where K_F_ ((mg/kg)/(mg/L)^1/nF^) is the Freundlich affinity coefficient, which is related to the adsorption capacity; n_F_ is a constant, 1/n_F_ is considered as an indicator of the strength of the adsorption group; Q_e_ (mg/kg) and C_e_ (mg/L) are the adsorption capacity on the solid phase and the equilibrium concentration in the suspension.

#### 2.2.3. Error Functions

The residual root mean square error (RMSE) and the average relative error (ARE) were employed in the error functions:

The RMSE is:

(5) $$\sqrt{\frac{1}{n-2}\overset{n}{\underset{i=1}{\sum }}{\left(Q_{e,exp}-Q_{e,cal}\right)}^{2}}$$

The ARE is:

(6) $$\overset{n}{\underset{i=1}{\sum }}\left|\frac{Q_{e,exp}-Q_{e,calc}}{Q_{e,exp}}\right|$$

where Q_e,exp_ is the observed adsorption capacity; Q_e,cal_ is the calculated values of different models; n is the number of observations in the experimental data.

The smaller the RMSE value and ARE value, the better the curve fitting [39].

#### 2.2.4. The Effects of pH on Sb Adsorption

Briefly, 200 mg/L Sb (III) and Sb (V) solutions were prepared at room temperature (25 °C). The pH of the solution was adjusted to 4, 7, and 10 with NaOH and HCl. Hence, 2.0000 g of soils were added to a 50 mL plastic centrifuge tube. The KCl concentration, soil/water ratio, and shaking time were the same as in Section 2.2.1.

### 2.3. Soil Analysis and Characterization

Soil pH was measured according to the standard method (NY-T 1377-2007, China) using a pH meter (S210-S, Mettler Toledo, Greifensee, Switzerland). Cation exchange capacity (CEC) was determined according to the standard method (NY-T 295-1995, China) using an atomic absorption spectrophotometer (PinAAcle 900 Z) to measure the absorbance of Ca and Mg, with the wavelength 422.7 nm 285.2 nm, respectively. Organic matter (OM) was measured according to the standard method (NY-T 1121.6-2006, China). The pore size, pore volume, and specific surface area of soil particles were measured using automatic specific surface and porosity analyzer BET (ASAP2460, Micromeritics Instrument Corp., Atlanta, GA, USA), respectively. Sb speciation was analyzed using Tessier sequential extraction. The particle sizes were measured using sieves and hydrometers (NY/T 1121.3-2006).

The samples were sprayed with gold and scanned by the electron microscope (ZEISS Gemini 300, Zeiss, Germany) with X-ray spectrometer (Oxford Xplore, Abingdon, UK). The pictures were used to observe the comparison of the surface morphology the Sb distribution of soil particles before and after adsorption. X-ray photoelectron spectroscopy (XPS) was used to detect the changes in surface Sb valence of soil particles before and after the absorption.

## 3. Results and Discussion

### 3.1. The Adsorption Behaviors of Sb onto Soils

#### 3.1.1. The Effects of Contact Times on Adsorption Behaviors of Sb

**Figure 1:**
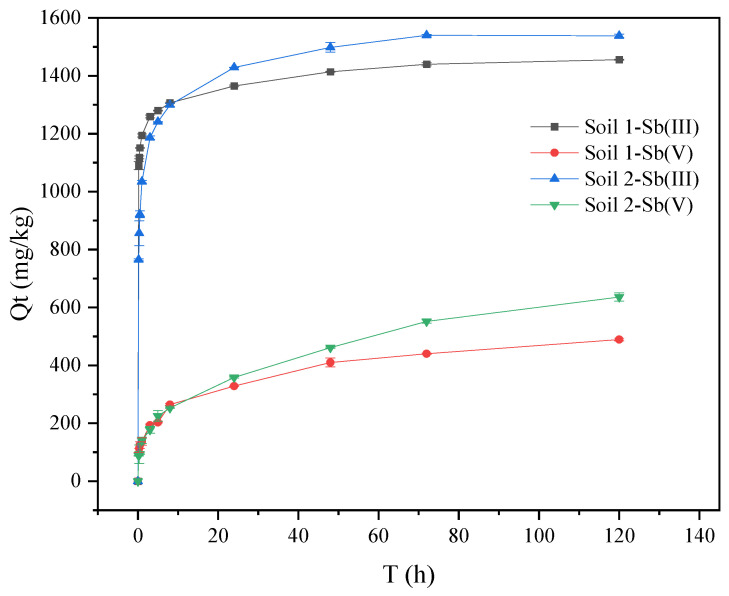
Variation of Sb adsorption on soils with time.

As shown in Figure 1, the adsorption capacity of Sb onto two soils increased rapidly with time within 8 h, which was in the fast adsorption stage. Then, the adsorption capacity of Sb increased slowly after 8 h. After 24 h, the adsorption of Sb (III) gradually reached equilibrium. After 72 h, the adsorption of Sb (V) gradually reached equilibrium. In addition, the adsorption rate of Sb onto soil 1 was greater than that of soil 2, but the adsorption capacity of Sb onto soil 1 was smaller than that of soil 2. The clay colloid percentage of soil 1 was higher, which means soil 1 had more specific surface area and adsorption sites, which led to a higher adsorption rate than soil 2 in the rapid adsorption stage [31,35]. The equilibrium adsorption capacity might be related to the presence of calcium oxide, aluminum oxide, iron oxide, and manganese oxide [34,40,41,42,43,44].

#### 3.1.2. The Effects of Initial Concentration on Adsorption Behaviors of Sb

**Figure 2:**
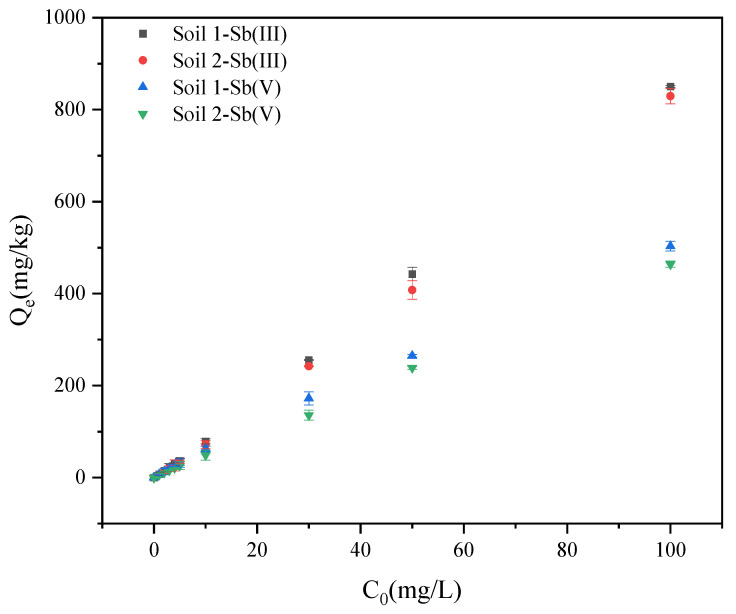
Variation of Sb adsorption on soil with initial concentration.

As shown in Figure 2, with the increase of the initial concentration, more ions appeared in the solution for the exchange reaction, which increased the adsorption capacity of the soils at equilibrium. The adsorption capacity of Sb (III) in soils was higher than that of Sb (V) at equilibrium. In addition, the difference between the equilibrium adsorption capacity of Sb (III) and Sb (V) gradually increased.

#### 3.1.3. The Effects of pH on Adsorption Behaviors of Sb on Soils

**Figure 3:**
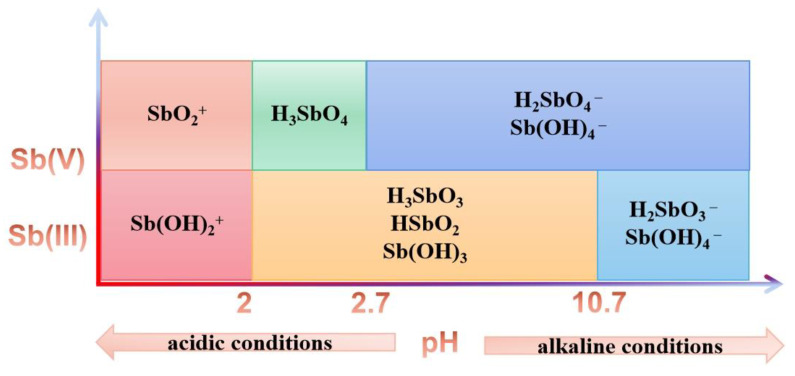
Chemical forms of Sb at different pH values [45].

As shown in Figure 3, Sb (III) existed as neutral molecules such as Sb (OH)_3_, H_3_SbO_3_, or HSbO_2_ in the pH range of 2–10.7 and existed as SbO^+^ or Sb (OH)_2_^+^ under strong acid conditions [45]. In a strong alkaline environment, it existed as SbO_2_^−^ or Sb (OH)_4_^−^, and only as Sb^3+^ under extremely acidic conditions. Sb (V) existed as Sb (OH)_6_^−^, H_2_SbO_4_^−^, or SbO_3_^−^ under weak acid, neutral, and alkaline, and it would exist in the form of SbO_2_^−^ under strong acid conditions.

**Figure 4:**
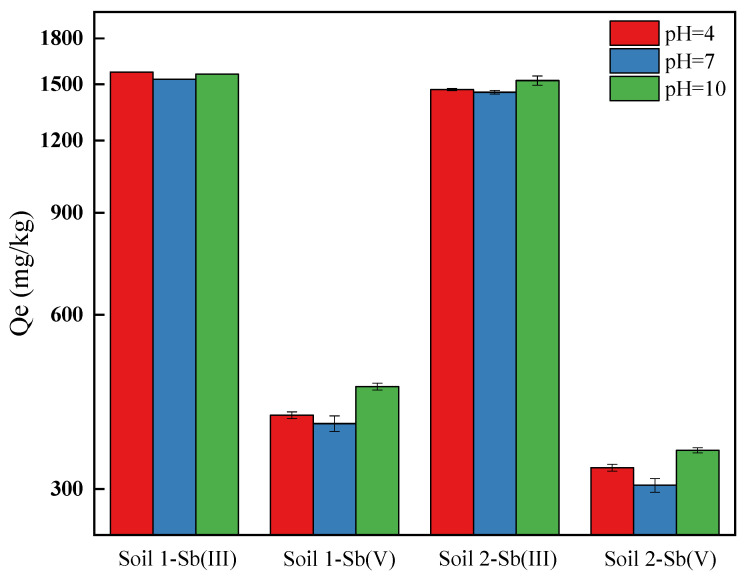
Adsorption equilibrium of Sb under different pH conditions.

The pH of groundwater is between mildly acidic to neutral. The adsorption capacities of Sb (III) and Sb (V) under pH = 4, 7, and 10 are shown in Figure 4. Under pH = 7, The adsorption capacities of Sb onto soils were lowest. When pH ranged between 4 and 10 in the solution, Sb (III) usually existed as neutral molecules, and the following reaction equilibrium existed in the solution [46]:

(7) $$Sb{\left(OH\right)}_{3\left(aq\right)}+H_{2}O_{\left(l\right)}↔Sb{\left(OH\right)}_{4\left(aq\right)}^{-}+H_{\left(aq\right)}^{+}$$

The experimental results of Sb (III) were better fitted using Langmuir and Freundlich isotherm adsorption models and pseudo first order kinetic model (Section 3.2), indicating that the adsorption processes were chemical adsorption. It has been shown that Sb (III) is strongly adsorbed on goethite over a pH range of 3–12 [36]. When pH increased from 4 to 10, the equilibrium adsorption capacity of Sb (III) did not change significantly, while it was relatively small in neutral solution, due to the low background ionic strength in the solution, while H^+^ and OH^−^ concentrations were low. Although Sb (III) existed in the form of stable neutral molecules, various factors, including colloidal particles, amorphous iron oxides, calcium oxides, and organic matter, were affected by pH value, which inhibited the adsorption reaction. In general, pH changes had little effect on Sb adsorption, which was related to the existence of Sb in the form of neutral molecules in the pH range of 2–10.7 [46].

Sb (V) existed in the anion forms of Sb (OH)_6_^−^, H_2_SbO_4_^−^, or SbO_3_^−^ at pH = 4, 7, and 10 [46]. The adsorption was usually due to diffusive limitations at the soil interface due to electrostatic repulsion, slow solid state diffusion of the initially metal adsorbed surface, slow surface precipitation and adsorption kinetic barriers, or a combination of the above [47]. As pH increased, the ion concentrations increased. In addition, the adsorption of Sb onto soil particles was more competitive with ions. On the other hand, the surface potential of soil particles decreased and the electrostatic repulsion increased with the decreasing of the electrostatic attraction of anions [48]. Therefore, when pH gradually increased, the equilibrium adsorption capacity of Sb (V) gradually decreased.

### 3.2. The Adsorption Mechanism of Sb onto Soils

#### 3.2.1. Adsorption Kinetic Experiments

**Figure 5:**
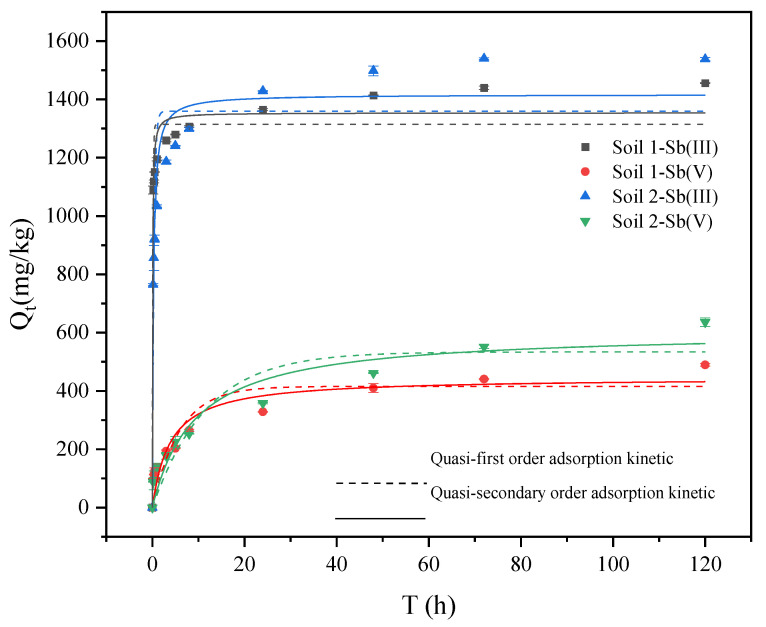
Sb (III) and Sb (V) adsorption kinetics fitting curve.

The results are shown in Table 2 and Figure 5. Through the error analysis, the ARE and RMSE values of the first order kinetic model were lower than those of the second order kinetic model. In the fitting first order kinetic model, R^2^ for Sb (III) was above 0.90, and R^2^ for Sb (V) was above 0.82. These results indicated that the first order kinetic model could better describe the adsorption of Sb onto soils. Previous studies showed that the adsorption of Sb onto kaolinite was consistent with the first order kinetic model and the second-order kinetic model [49]. In Figure 5, the adsorption of Sb is mainly divided into a fast and slow adsorption stage, respectively. The adsorption of Sb (III) and Sb (V) onto the surface of soil mineral colloids was related to the surface hydroxyl groups, which combined with oxygen to form internal spherical complexes [40,50,51]. At the beginning of the adsorption, the initial concentration of Sb was high, and the soils had more adsorption sites, which led to the high adsorption rate. As the reaction progressed, the adsorption sites in soils decreased, the adsorption process gradually became a diffusion process, and thus the adsorption rate decreased [52]. Within 8 h of the adsorption, the adsorption rates of Sb (III) and Sb (V) by soil 1 were higher than that of soil 2. The differences might be related to soil texture. Soil 1 had smaller particles, larger specific surface areas, more adsorption sites, and high organic matter contents, leading to a faster adsorption. The adsorption reaction of Sb (III) and Sb (V) reached equilibrium within 48 h, but the time for Sb (III) to reach equilibrium was shorter. The equilibrium adsorption capacities of Sb (III) in soil 1 and soil 2 were 1314.46 mg/kg and 1359.25 mg/kg, respectively, while the equilibrium adsorption capacities of Sb (V) were 415.65 mg/kg and 553.97 mg/kg, respectively. Besides that, the limiting factor of the Sb adsorption rate was related to the diffusion of adsorbate molecules on the interface. The electrostatic attraction and repulsion on the surface of the adsorbent were related to the potential binding capacity of the adsorbent surface and the surface chemical reaction.

#### 3.2.2. Adsorption Isotherm Experiments

**Figure 6:**
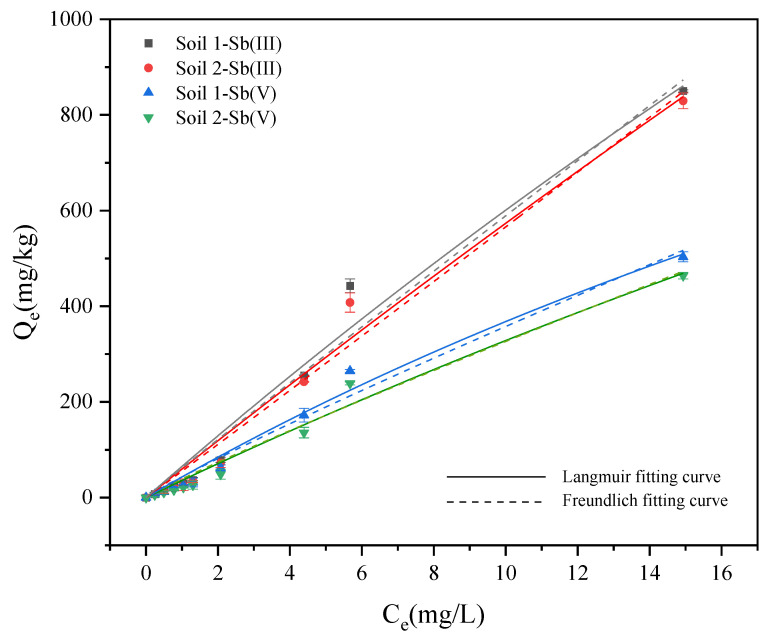
Fitting curve of Sb (**III**) and Sb (**V**) adsorption isotherm.

The results are shown in Table 3 and Figure 6. In general, the adsorption isotherm describes how adsorbates interact with adsorbents. The isotherm described by the Langmuir model was a progressive line that gradually tended to be gentle with the increase of concentration. The adsorption surface was assumed to be the ideal smooth surface, and only single layer adsorption occurred. This model could describe chemical adsorption well [53]. Different from the Langmuir model, the isotherm described by the Freundlich model showed an infinitely rising trend, and it was assumed that the adsorbent surface was heterogeneous and the active adsorption sites were unevenly distributed [53]. Figure 6 shows the Langmuir and Freundlich adsorption isotherms of Sb (III) and Sb (V). Table 1 shows the fitting parameters of Sb (III) and Sb (V) adsorption isotherms. Both models had good fitting results on Sb (III) and Sb (V), and R^2^ was above 0.97. Through the error analysis, the ARE and RMSE values of the Langmuir model were similar to the Freundlich model. The K_L_ value of soil 1 was greater than that of soil 2, and the K_L_ value of Sb (III) was greater than that of Sb (V). The results indicated that two soil samples have good affinity for Sb (III). The K_F_ value in the Freundlich model was related to the adsorption affinity, which indicated that the larger the K_F_ value, the better the adsorption effect [54]. The K_F_ value of soil 1 was greater than that of soil 2, and the K_F_ value of Sb (III) was greater than that of Sb (V). In addition, 1/n_F_ was slightly less than 1, which belonged to the “L-shaped” adsorption isotherm, which implied that the adsorption capacity increased with the equilibrium concentration and finally tended to equilibrium [55]. For Sb, it was a multilayer adsorption on heterogeneous surfaces with different affinities on the soils. Anastasia reported similar results in that the adsorption data of Sb (III) and Sb (V) onto hydrous Al Oxide and clay minerals including kaolinite could be well fitted with either Freundlich or Langmuir isotherms [40].

### 3.3. The Transformation of Chemical Species of Sb in Soils

**Figure 7:**
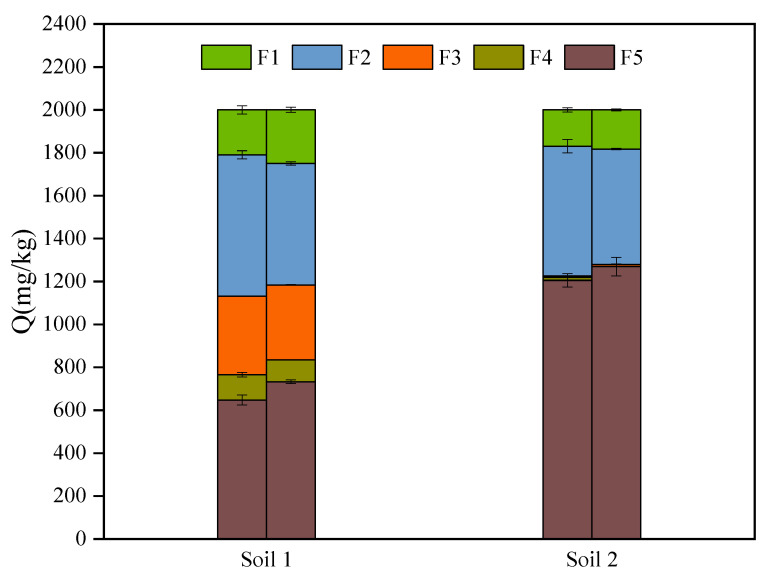
Sequential extraction by Tessier methods for soils after adsorption.

The Tessier sequential extraction results are shown in Figure 7. Previous studies showed that the water soluble, carbonate-bound, and exchangeable fractions of heavy metals can be directly absorbed and utilized by plants [56]. The organic -bound fractions cannot be easily used and the residual fractions were almost unusable by organisms. After being absorbed for 120 h in soil 2, Sb (III) and Sb (V) mainly existed in the residual fractions, followed by carbonate-bound and water-soluble fractions. Organic matter contents in soil 2 were less than 0.5%, while in soil 1 they were about 2.5%. The proportion of carbonate-bound and water-soluble fractions of Sb in soil 1 was similar to soil 2, but its percentages of organic-bound and Fe/Mn-bound fractions in soil 1 were higher than in soil 2. Moreover, SEM/EDS results indicated that iron and manganese were not detected in soil 2 but could be determined in soil 1. The following reactions might occur [46]:

(8) $$2Fe{\left(OH\right)}_{3\left(s\right)}+Sb{\left(OH\right)}_{3\left(aq\right)}\to 2Fe{\left(OH\right)}_{2\left(s\right)}+H_{3}SbO_{4\left(aq\right)}+H_{2}O_{\left(l\right)}$$

### 3.4. Insight into the Adsorption Behaviors of Sb on Soils

**Figure 8:**
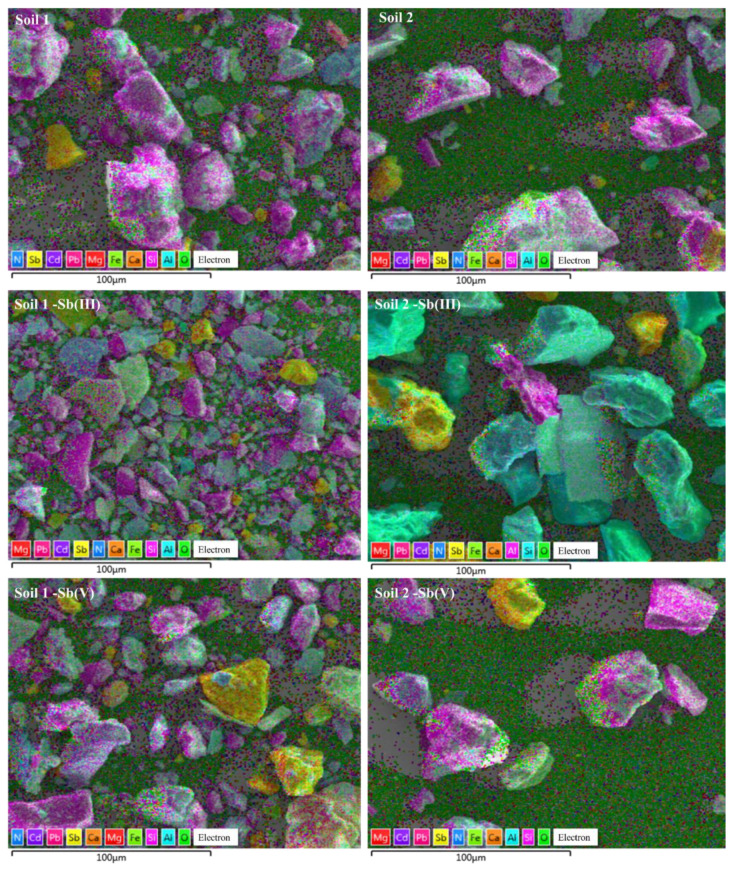
Scanning Electron Microscope (SEM) mapping of Sb on soils.

Under a soil/water ratio of 1:10 and the initial Sb concentration of 2000 mg/L, the element distribution on soil surfaces scanned by SEM mapping are shown in Figure 8.

It can be seen that the particles of soil 1 in the original soil were significantly smaller than those of soil 2. In addition, the contents of Al and Si in soils were relatively high with a lower content of Ca, Mg, Fe, and Sb. The distribution position of Sb in the original soil coincided with Ca. In addition, those particles might be made up of calcium antimonite minerals, such as Ca [Sb (OH)_6_]_2_ and Ca_2_Sb_2_O_7_.

After the adsorption of Sb (III) and Sb (V), it could be clearly seen that the Sb was more sporadically distributed and its distribution areas increased, indicating that Sb was adsorbed onto the soils. Similar to the original soil, the distribution areas of Sb in the Sb adsorbed soils still highly overlapped with the distribution of Ca and had no obvious association with the distribution of Fe, Al, and Mg. The results can be explained by the following chemical adsorption reaction [57,58]:

(9) $$\mathrm{Sb}{\left(\mathrm{OH}\right)}_{3}+3H_{2}O=2e^{-}+3H^{+}+\mathrm{Sb}{\left(\mathrm{OH}\right)}_{6}^{-}$$

(10) $$Ca^{2+}+Sb{\left(OH\right)}_{6}^{-}=CaSb{\left(OH\right)}_{6}^{+}$$

**Figure 9:**
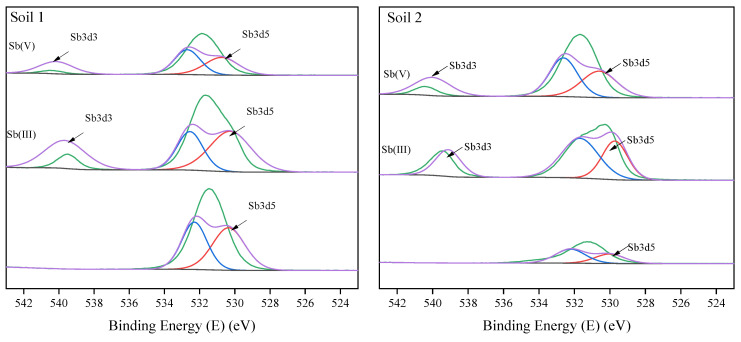
XPS spectra of the soil samples before and after adsorption.

To better understand the adsorption behavior of Sb, its surface chemical binding states in soils were analyzed through XPS results. It was clearly seen from Figure 9 that before Sb (III) and Sb (V) adsorption, XPS spectrums of soil 1 and soil 2 could be divided into Sb3d5 at the peak of 530.7 ev. After the adsorption, the XPS spectrum of the soils could be deconvoluted into Sb3d3 and Sb3d5, representing Sb (III) and Sb (V) at the peaks of 530.7 ev and 540.1 ev. The results implied that the redox reactions might accompany the changes in Sb valences during the adsorption. The reduction of Sb (V) to Sb (III) in soils has been shown to occur with the ferrous iron [59], while the oxidation is enhanced by Fe/Mn oxyhydroxides present in natural soils [46] and the reactive oxygen species (ROS), including hydroxyl (·OH), peroxyl radicals (·ROO), hydrogen peroxide (H_2_O_2_), and superoxide radical anion (·O_2_^−^) in groundwater [60]. Besides, pH had an effect on the redox rate of Sb [31,35,36].

### 3.5. How to Understand the Migration and Transformation of Sb between Soil and Groundwater

Naturally occurring geochemical processes in mineral phases associated with rock, soils, and sediments of unsaturated zones and aquifers are directly linked with contamination levels and the migration of Sb in the soil and groundwater system [46]. Sb is usually adsorbed and desorbed in soil and groundwater and achieves valence transition through redox on the surface of soil particles [61]. The transformation of mobile forms of Sb is predominantly controlled by naturally occurring adsorption and precipitation processes. Organic matter, soil colloid, as well as iron, aluminum, and manganese oxyhydroxides have been recognized as naturally occurring Sb sequestrating agents in the environment. Sb mobility is also affected by its co-precipitation with alkali metals, which results in the formation of stable mineral phases, such as calcium antimonates and tripuhyite [34,40,41,42,43,44]. In Ca rich environmental mediums, such as calcareous soils and alkaline waste materials, calcium antimonates (Ca_1+x_Sb_2_O_6_OH_2−2x_ and Ca[Sb(OH)_6_]_2_) as the main chemical composition of roméite minerals are suggested as an important sink for Sb [62]. Moreover, in some industrial waste systems, the solubility product of a hydrated calcium antimonate indicated the formation of Sb bearing precipitates [63]. These mineral phases can further prevent Sb migrating from shallower to deeper strata. Thus, it was difficult for Sb to follow groundwater to migrate over long distances. From the previous studies above, Sb sequestered in a solid might form on calcite surfaces through the precipitation process in two soils, which contributes to the majority of the whole Sb sequestration. The interface reactions can be schematically represented as follows [42]:

(11) $$CaCO_{3}+H^{+}\to \;Ca^{2+}+HCO_{3}^{-}$$

(12) $$Ca^{2+}+Sb^{\left(v\right)}\to Ca-Sb^{\left(v\right)}$$

## 4. Conclusions

Sb pollution in soils and groundwater pose a threat to human health. Thus, a better understanding of the mobility, fate, and transportation of Sb in soils and groundwater is crucial to establishing a sustainable Sb mitigation on a regional scale. In this study, the adsorption characteristics of Sb in two soils were fitted using two adsorption kinetic models and two isotherm adsorption models. Among these, the first order kinetic model fitted the better kinetic results with the analysis of R^2^ and error functions. In addition, the maximum adsorbed amount of Sb was found to be close to the actual values. The adsorption isotherm could be better described by Langmuir and Freundlich models, revealing that the reaction process was a chemisorption. Besides, pH has little effect on Sb adsorption. Combined with SEM and XPS analysis, the result showed that the adsorption behavior of Sb onto soils was mainly related to Ca bearing minerals, which resulted in the formation of calcium antimonates. This work may provide new insight into Sb adsorption onto soils, as well as a theoretical guide for studies on the adsorption and migration behavior of Sb in the soil and groundwater system.

## Figures and Tables

**Table 1:** Physical and chemical properties of the studied soil samples.

Soil Categories	Soil 1	Soil 2
pH	8.19	8.61
CEC (cmol/kg)	47.35	14.45
SOM (g/kg)	24.96	4.53
BET surface area (m^2^/g)	14.07	6.52
Pore volume (cm^3^/g)	0.0199	0.0117
Pore size (nm)	5.646	7.172
Particle sizes	<0.002 mm 45.3% 0.002–0.02 mm 50.1% >0.02 mm 4.6%	<0.002 mm 1% 0.002–0.02 mm 13.3% >0.02 mm 85.7%

**Table 2:** Sb (III) and Sb (V) adsorption kinetic model parameters.

Soil Sample	Valence State	First Order Kinetic Model					Second Kinetic Model				
		Q_e_	k_1_	R^2^	ARE	RMSE	Q_e_	k_2_	R^2^	ARE	RMSE
soil 1	Sb (III)	1314.46	8.72	0.934	0.782	95.95	1354.07	0.013	0.969	0.927	118.66
	Sb (V)	415.65	0.17	0.828	3.875	61.15	445.69	5.76 × 10^−4^	0.887	6.955	129.77
soil 2	Sb (III)	1359.25	2.88	0.872	1.370	150.69	1416.86	0.003	0.943	2.364	257.30
	Sb (V)	533.97	0.09	0.870	4.380	69.80	606.06	1.78 × 10^−4^	0.916	9.733	177.77

**Table 3:** Sb (III) and Sb (V) isothermal adsorption fitting parameters.

Soil Categories	Valence State	Langmuir Model					Freundlich Model				
		Q_m_	K_L_	R^2^	ARE	RMSE	K_F_	n_F_	R^2^	ARE	RMSE
soil 1	Sb (III)	6949.72	0.009	0.975	7.192	40.52	61.55	1.019	0.974	6.522	42.23
	Sb (V)	2285.78	0.019	0.984	3.991	18.76	43.26	1.090	0.981	4.292	21.50
soil 2	Sb (III)	12981.87	0.005	0.979	7.529	36.13	55.21	0.989	0.979	6.483	36.58
	Sb (V)	3564.77	0.010	0.980	4.396	19.41	38.88	1.082	0.999	5.476	21.05

## Data Availability

All data generated or analyzed during this study are included in this published article.

## References

[1] Li J., Wang Q., Li M., Yang B., Shi M., Guo W., McDermott T.R., Rensing C., Wang G. (2015). Proteomics and Genetics for Identi-fication of a Bacterial Antimonite Oxidase in *Agrobacterium tumefaciens*. Environ. Sci. Technol..

[2] Okkenhaug G., Zhu Y.-G., Luo L., Lei M., Li X., Mulder J. (2011). Distribution, speciation and availability of antimony (Sb) in soils and terrestrial plants from an active Sb mining area. Environ. Pollut..

[3] Wu F., Fu Z., Liu B., Mo C., Chen B., Corns W., Liao H. (2011). Health risk associated with dietary co-exposure to high levels of antimony and arsenic in the world’s largest antimony mine area. Sci. Total Environ..

[4] Wang Q., He M., Wang Y. (2011). Influence of combined pollution of antimony and arsenic on culturable soil microbial populations and enzyme activities. Ecotoxicology.

[5] An Y.-J., Kim M. (2009). Effect of antimony on the microbial growth and the activities of soil enzymes. Chemosphere.

[6] Bhattacharyya P., Tripathy S., Kim K., Kim S.-H. (2008). Arsenic fractions and enzyme activities in arsenic-contaminated soils by groundwater irrigation in West Bengal. Ecotoxicol. Environ. Saf..

[7] Bordeleau G., Martel R., Ampleman G., Thiboutot S. (2008). Environmental Impacts of Training Activities at an Air Weapons Range. J. Environ. Qual..

[8] Gebel T. (1997). Arsenic and antimony: Comparative approach on mechanistic toxicology. Chem. Interact..

[9] Chang C., Li F., Wang Q., Hu M., Du Y., Zhang X., Zhang X., Chen C., Yu H.-Y. (2022). Bioavailability of antimony and arsenic in a flowering cabbage–soil system: Controlling factors and interactive effect. Sci. Total Environ..

[10] Li Y., Lin H., Gao P., Yang N., Xu R., Sun X., Li B., Xu F., Wang X., Song B. (2021). Variation in the diazotrophic community in a vertical soil profile contaminated with antimony and arsenic. Environ. Pollut..

[11] Zhong Q., Li L., He M., Ouyang W., Lin C., Liu X. (2021). Toxicity and bioavailability of antimony to the earthworm (*Eisenia fetida*) in different agricultural soils. Environ. Pollut..

[12] Ashley P.M., Craw D., Tighe M.K., Wilson N.J. (2006). Magnitudes, spatial scales and processes of environmental antimony mobility from orogenic gold–antimony mineral deposits, Australasia. Environ. Earth Sci..

[13] Henckens M.L.C.M., Driessen P.P.J., Worrell E. (2016). How can we adapt to geological scarcity of antimony? Investigation of antimony’s substitutability and of other measures to achieve a sustainable use. Resour. Conserv. Recycl..

[14] McNamara D.D., Sewell S., Buscarlet E., Wallis I.C. (2016). A review of the Rotokawa Geothermal Field, New Zealand. Geothermics.

[15] Simmons S.F., Brown K.L., Browne P.R., Rowland J.V. (2016). Gold and silver resources in Taupo Volcanic Zone geothermal systems. Geothermics.

[16] Wang X., He M., Xi J., Lu X. (2011). Antimony distribution and mobility in rivers around the world’s largest antimony mine of Xikuangshan, Hunan Province, China. Microchem. J..

[17] Wu Y., Liu Q., Ma J., Zhao W., Chen H., Qu Y. (2021). Antimony, beryllium, cobalt, and vanadium in urban park soils in Beijing: Machine learning-based source identification and health risk-based soil environmental criteria. Environ. Pollut..

[18] Busby R.R., Barbato R.A., Jung C.M., Bednar A.J., Douglas T.A., Ringelberg D.B., Indest K.J. (2021). Alaskan plants and their assembled rhizosphere communities vary in their responses to soil antimony. Appl. Soil Ecol..

[19] He M., Wan H. (2004). Distribution, speciation, toxicity and bioavailability of antimony in the environment. Prog. Chem..

[20] Rong Q., Nong X., Zhang C., Zhong K., Zhao H. (2022). Immobilization mechanism of antimony by applying zirconium-manganese oxide in soil. Sci. Total Environ..

[21] Xia B., Yang Y., Li F., Liu T. (2022). Kinetics of antimony biogeochemical processes under pre-definite anaerobic and aerobic con-ditions in a paddy soil. J. Environ. Sci..

[22] He Z., Liu R., Liu H., Qu J. (2015). Adsorption of Sb(III) and Sb(V) on Freshly Prepared Ferric Hydroxide (FeOxHy). Environ. Eng. Sci..

[23] Ilgen A., Majs F., Barker A., Douglas T., Trainor T. (2014). Oxidation and mobilization of metallic antimony in aqueous systems with simulated groundwater. Geochim. Et Cosmochim. Acta.

[24] Takaoka M., Fukutani S., Yamamoto T., Horiuchi M., Satta N., Takeda N., Oshita K., Yoneda M., Morisawa C., Tanaka T. (2005). De-termination of chemical form of antimony in contaminated soil around a smelter using X-ray absorption fine structure. Anal. Sci..

[25] Scheinost A.C., Rossberg A., Vantelon D., Xifra I., Kretzschmar R., Leuz A.-K., Funke H., Johnson C.A. (2006). Quantitative antimony speciation in shooting-range soils by EXAFS spectroscopy. Geochim. Et Cosmochim. Acta.

[26] Pilarski J., Waller P., Pickering W. (1995). Sorption of antimony species by humic acid. Water Air Soil Pollut..

[27] Lintschinger J., Michalke B., Schulte-Hostede S., Schramel P. (1998). Studies on Speciation of Antimony in Soil Contaminated by Industrial Activity. Int. J. Environ. Anal. Chem..

[28] Mitsunobu S., Harada T., Takahashi Y. (2006). Comparison of Antimony Behavior with that of Arsenic under Various Soil Redox Conditions. Environ. Sci. Technol..

[29] Iqbal M., Saeed A., Edyvean R.G. (2013). Bioremoval of antimony(III) from contaminated water using several plant wastes: Optimization of batch and dynamic flow conditions for sorption by green bean husk (*Vigna radiata*). Chem. Eng. J..

[30] Tahervand S., Jalali M. (2017). Sorption and desorption of potentially toxic metals (Cd, Cu, Ni and Zn) by soil amended with bentonite, calcite and zeolite as a function of pH. J. Geochem. Explor..

[31] Xi J., He M., Lin C. (2009). Adsorption of antimony(V) on kaolinite as a function of pH, ionic strength and humic acid. Environ. Earth Sci..

[32] Brannon J., Patrick W. (1985). Fixation and mobilization of antimony in sediments. Environ. Pollut. Ser. B Chem. Phys..

[33] Zhang D., Guo J., Xie X., Zhang Y., Jing C. (2021). Acidity-dependent mobilization of antimony and arsenic in sediments near a mining area. J. Hazard. Mater..

[34] Jiang Y., Xia T., Jia X., Zhong M., Wang N., Li N. (2020). Study on stabilization of antimony(Sb)in contaminated soil by primary explosives using iron-based and aluminum-based adsorbents. China Environ. Sci..

[35] Rakshit S., Sarkar D., Datta R. (2015). Surface complexation of antimony on kaolinite. Chemosphere.

[36] Leuz A.-K., Mönch H., Johnson C.A. (2006). Sorption of Sb(III) and Sb(V) to Goethite: Influence on Sb(III) Oxidation and Mobilization. Environ. Sci. Technol..

[37] Do X.-H., Lee B.-K. (2013). Removal of Pb2+ using a biochar–alginate capsule in aqueous solution and capsule regeneration. J. Environ. Manag..

[38] Tavakoli H., Sepehrian H., Cheraghali R. (2013). Encapsulation of nanoporous MCM-41 in biopolymeric matrix of calcium alginate and its use as effective adsorbent for lead ions: Equilibrium, kinetic and thermodynamic studies. J. Taiwan Inst. Chem. Eng..

[39] Hadi M., Samarghandi M.R., McKay G. (2010). Equilibrium two-parameter isotherms of acid dyes sorption by activated carbons: Study of residual errors. Chem. Eng. J..

[40] Ilgen A.G., Trainor T.P. (2011). Sb(III) and Sb(V) Sorption onto Al-Rich Phases: Hydrous Al Oxide and the Clay Minerals Kaolinite KGa-1b and Oxidized and Reduced Nontronite NAu-1. Environ. Sci. Technol..

[41] Liu R., Xu W., He Z., Lan H., Liu H., Qu J., Prasai T. (2015). Adsorption of antimony(V) onto Mn(II)-enriched surfaces of manganese-oxide and Fe Mn binary oxide. Chemosphere.

[42] Renard F., Putnis C.V., Montes-Hernandez G., King H.E., Breedveld G.D., Okkenhaug G. (2018). Sequestration of Antimony on Calcite Observed by Time-Resolved Nanoscale Imaging. Environ. Sci. Technol..

[43] Guo X., Wu Z., He M., Meng X., Jin X., Qiu N., Zhang J. (2014). Adsorption of antimony onto iron oxyhydroxides: Adsorption behavior and surface structure. J. Hazard. Mater..

[44] Tighe M., Lockwood P., Wilson S. (2005). Adsorption of antimony(V) by floodplain soils, amorphous iron(III) hydroxide and humic acid. J. Environ. Monit..

[45] Dousova B., Buzek F., Herzogova L., Machovič V., Lhotka M. (2014). Effect of organic matter on arsenic(V) and antimony(V) adsorption in soils. Eur. J. Soil Sci..

[46] Herath I., Vithanage M., Bundschuh J. (2017). Antimony as a global dilemma: Geochemistry, mobility, fate and transport. Environ. Pollut..

[47] Verbeeck M., Moens C., Gustafsson J.P. (2021). Mechanisms of antimony ageing in soils: An XAS study. Appl. Geochem..

[48] Morel M.C., Spadini L., Brimo K., Martins J.M. (2014). Speciation study in the sulfamethoxazole-copper-pH-soil system: Implications for retention prediction. Sci. Total Environ..

[49] Xi J., He M., Kong L. (2016). Adsorption of antimony on kaolinite as a function of time, pH, HA and competitive anions. Environ. Earth Sci..

[50] Mittal V.K., Bera S., Narasimhan S., Velmurugan S. (2013). Adsorption behavior of antimony(III) oxyanions on magnetite surface in aqueous organic acid environment. Appl. Surf. Sci..

[51] Mason S.E., Trainor T.P., Goffinet C.J. (2012). DFT study of Sb(III) and Sb(V) adsorption and heterogeneous oxidation on hydrated oxide surfaces. Comput. Theor. Chem..

[52] Kadirvelu K., Namasivayam C. (2003). Activated carbon from coconut coirpith as metal adsorbent: Adsorption of Cd(II) from aqueous solution. Adv. Environ. Res..

[53] Xi J., He M., Lin C. (2011). Adsorption of antimony(III) and antimony(V) on bentonite: Kinetics, thermodynamics and anion competition. Microchem. J..

[54] Ho Y.S., Porter J.F., McKay G. (2002). Equilibrium Isotherm Studies for the Sorption of Divalent Metal Ions onto Peat: Copper, Nickel and Lead Single Component Systems. Water Air Soil Pollut..

[55] Singh N. (2002). Sorption Behavior of Triazole Fungicides in Indian Soils and Its Correlation with Soil Properties. J. Agric. Food Chem..

[56] Dai Y., Nasir M., Zhang Y., Wu H., Guo H., Lv J. (2017). Comparison of DGT with traditional methods for assessing cadmium bioavailability to Brassica chinensis in different soils. Sci. Rep..

[57] Cornelis G., Van Gerven T., Snellings R., Verbinnen B., Elsen J., Vandecasteele C. (2011). Stability of pyrochlores in alkaline matrices: Solubility of calcium antimonate. Appl. Geochem..

[58] Filella M., May P.M. (2003). Computer simulation of the low-molecular-weight inorganic species distribution of antimony(III) and an-timony(V) in natural waters. Geochim. Et Cosmochim. Acta.

[59] Kirsch R., Scheinost A.C., Rossberg A., Banerjee D., Charlet L. (2008). Reduction of antimony by nano-particulate magnetite and macki-nawite. Mineral. Mag..

[60] Zhang Y., O’Loughlin E.J., Kwon M.J. (2022). Antimony redox processes in the environment: A critical review of associated oxidants and reductants. J. Hazard. Mater..

[61] Cai Y., Mi Y., Zhang H. (2016). Kinetic modeling of antimony(III) oxidation and sorption in soils. J. Hazard. Mater..

[62] Brugger J., Gieré R., Graeser S., Meisser N. (1997). The crystal chemistry of roméite. Contrib. Mineral. Petrol..

[63] Cornelis G., Johnson C.A., Van Gerven T., Vandecasteele C. (2008). Leaching mechanisms of oxyanionic metalloid and metal species in alkaline solid wastes: A review. Appl. Geochem..

